# Predictors of the Health-Related Quality of Life (HRQOL) in SF-36 in Knee Osteoarthritis Patients: A Multimodal Model With Moderators and Mediators

**DOI:** 10.7759/cureus.27339

**Published:** 2022-07-27

**Authors:** Sara Pinto Barbosa, Lucas Marques, Andre Sugawara, Fernanda Toledo, Marta Imamura, Linamara Battistella, Marcel Simis, Felipe Fregni

**Affiliations:** 1 Clinical Research Center of the Institute of Physical Medicine and Rehabilitation of Hospital das Clínicas – FMUSP, Faculdade de Medicina FMUSP, Universidade de Sao Paulo, São Paulo, BRA; 2 Hospital das Clínicas, Faculdade de Medicina FMUSP, Universidade de Sao Paulo, São Paulo, BRA; 3 Clinical Research Center of the Institute of Physical Medicine and Rehabilitation of Hospital das Clínicas – FMUSP, Faculdade de Medicina FMUSP, Universidade de Sao Paulo, Sao Paulo, BRA; 4 Neuromodulation Center and Center for Clinical Research Learning, Spaulding Rehabilitation Hospital and Massachusetts General Hospital, Harvard Medical School, Boston, USA

**Keywords:** rehabilitation, physical function, pain, knee osteoarthritis, sf-36, quality of life

## Abstract

Purpose

The study aimed to examine associations between the 36-item short form health survey (SF-36) in clinical and neurophysiological measures to identify its predictors in patients with knee osteoarthritis (KOA) in a rehabilitation program.

Methods

We analyzed data from our cohort study (DEFINE cohort). We analyzed data from our KOA arm, with 107 patients, including clinical assessments, demographic data, pain scales, motor function (Timed Up and Go Test (TUG), 10 meters walk test, and 6-minute walk), balance (BBS), sleepiness (ESS), and Transcranial Magnetic Stimulation (TMS) and Electroencephalography (EEG).

Results

Our results showed 83.19% of patients were female with an average age of 68.6 years and an average number of days of pain was 96 days; around 31.86% were using more than five medications per day. Regarding the multimodal model to explain SF-36, the main variables relevant to the quality of life (QoL) were related to emotional aspects, such as anxiety and depression. Moreover, our study added findings with polymorphism (OPRM1/rs1799971) predicting mental aspects. Cognitive variables were important in predicting the mental health, emotional, and social support dimensions of the SF-36. In the physical domain, pain-related variables predominantly predicted QoL in these relationships. The domain of vitality significantly predicted all dimensions studied, except for mental and general health.

Conclusion

The results help in understanding the aspects that contribute to QoL and are discussed considering the general literature on physical rehabilitation and specific to this clinical group. Furthermore, the statistical methods allowed us to explore and effectively understand the dimensions related to QoL.

## Introduction

Osteoarthritis is a cartilage disease caused by multifactorial aspects with little understanding of the molecular mechanisms of initiation and progression [1]. Moreover, it is a degenerative joint disease that can compromise the knee and cause changes in bone, synovium, ligament, muscle, and periarticular fat [2]. Among adults aged 60 years and older, 10% of men and 13% of women are affected [3]. The clinical aspects have a clear and important impact on quality of life (QoL), mainly due to pain, stiffness, decreased mobility, loss of flexibility, and inflammation, leading to disability around the world and limiting patients to walking, stair climbing, squatting, and significantly changes daily activities and recreation [4]. It is known that the common symptoms are knee pain that is gradual in onset and worsens with activity and leads to conservative treatment until it progresses to surgical treatment options when conservative treatment fails [5].

Knee osteoarthritis (KOA) clinical symptoms vary among individuals but typically become severe, frequent, and more debilitating over time. Although medications help to slow the progression, there are no proven agents for the treatment [5], and it is sufficient to highlight the role of the interprofessional team and the necessity to assess as many aspects of patients with KOA, including health-related QoL aspects.

Although in 2001, a science conference, organized and sponsored by the Kessler Medical Rehabilitation Research and Education Corporation, in conjunction with federal and private, set QoL as a measure of rehabilitation, it is important to better understand the benefits of rehabilitation [6]. Moreover, although 20 years is not sufficiently debated in the literature, the understanding of the aspects related to QoL in people with KOA can provide us with a way to intervene and plan rehabilitation. Assessing QoL in rehabilitation patients is recommended by the World Health Organization [7] and this can be done using the Medical Outcomes Study Short Form 36 (SF-36) questionnaire.

As QoL cannot be directly measured, but under one aspect, the health-related quality of life (HRQOL) as a subset, it usually is converted into components that can be quantified [8]. Thus, the Short Form Health Survey is a questionnaire with 36 item (known as SF-36) to quantify some health domains, and could be assessed by eight subscales: 1) physical functioning, 2) physical role limitations, 3) emotional role limitations, 4) vitality, 5) mental health, 6) social functioning, 7) bodily pain, and 8) general health perceptions, which includes questions related to one’s health judgment [9]. Each dominion has a different distribution for the 36 questions regarding the measures and organization. The SF-36 is a QoL scale widely used in health studies [10].

For easy interpretation, a higher score indicates better health status [9]. Similarly, we can analyze the SF-36 through two dominions: physical (encompassing functional capacity, general health status, pain, and physical aspects) and mental (including mental health, vitality, social aspects, and emotional domains). This questionnaire was well designed for use in research and clinical practice and has been useful in health policy evaluations and general population surveys [11].

The physical functioning dimension questions aimed to measure the impact of physical limitations of everyday life on HRQOL. The questions focused on aspects, such as reduction of the amount of work or other activities, limitations of activities performed, and reduced accomplishments resulting from physical limitations. Those on emotional role limitations were about the reduction of work or daily activities due to emotional problems. In vitality, dominions have questions about energy and fatigue levels.

The mental health dominion questions were about happiness, cheer, peace, and negative affect for the last two weeks. Social aspects capture social activities regarding quantity and quality activities and the impact of physical and emotional problems on them. Regarding mental health, the questions include concepts of anxiety, depression, loss of behavioral or emotional control, and psychological well-being, which inspire us to be careful about potential risk to overlapping mainly to analyze mental health impact [12] in our results model. The bodily pain dimension measures the intensity and discomfort caused by the pain and interference in normal work in the last two weeks. General health perceptions provide a holistic perception of health, understanding current perception, resistance to disease, and healthy appearance [13].

In Brazil, the SF-36 was standardized by Laguardia et al. [14], and it showed scores below other countries, except general health and vitality. However, it is important to highlight that in the rehabilitated population, there are no studies with a considerable sample size in Brazil.

Hence, it is important to know the aspects that most influence the domains that are related to QoL to be more assertive during rehabilitation and related aspects to promote a positive impact on QoL. Therefore, the purpose of this study is to identify predictors of SF-36 in KOA patients in a rehabilitation program.

## Materials and methods

The data are obtained from a cohort study whose protocol was previously published by Simis et al. [13]. We focused on analyzing the cross-sectional data of the KOA group.

Sampling methods and participants 

The sample size of 107 patients was determined by the fact that the main design of the study was observational. Participants were recruited from the rehabilitation program at the Lucy Montoro Rehabilitation Institute (LMRI), which is in the city of São Paulo, as part of the Institute of Physical Medicine and Rehabilitation (IPMR) of the Clinics Hospital of the University of São Paulo Medical School. We included patients aged over 50 years with clinical and radiological diagnosis (Kellgren and Lawrence) of primary osteoarthritis of the knee and gonalgia for three months or more. Those with the following characteristics were excluded: i) pregnancy; ii) active osteoarthritis with clinical manifestations other than the knee; and iii) occurrence of other clinical and/or social conditions that hinder the subject's participation in the rehabilitation treatment. All participants were included from December 2018 to January 2020, and a total of 113 participants completed the study. Notably, all participants were evaluated before the global pandemic crisis, so the results found do not represent the scenarios during COVID-19.

Screening and assessment of severity

The patients went through clinical assessment and screening. All screenings were conducted by physicians trained to diagnose KOA, and they performed radiological bilateral knees. The visual analog scale (VAS), timed up and go (TUG), Berg Balance Scale (BBS), Western Ontario and McMasters University Osteoarthritis Index (WOMAC), Epworth sleepiness scale (ESS), and 10 meters walk test along with sociodemographic data collection and use of medications were performed by a physical therapist from the research center with previous experience. The Pain Catastrophizing Scale, McGill scale, Hamilton, hospital anxiety and depression (HAD) scale, and Montreal cognitive assessment (MoCA) were used by psychologists. To assess the severity of KOA, Kellegren-Lawrence was used in both knees where the radiograph had a grade (0 to 4), which correlated with increasing severity of KOA with grade 0 referring to no presence of OA, and grade 4 signifying severe OA. A detailed and complete description of each investigated instrument can be found in Simis et al. [15].

Neurophysiological markers

Following the study protocol of this project [15], TMS measures such as motor threshold (MT), cortical silent period (CSP), intracortical inhibition (SICI), intracortical facilitation (ICF), and motor evoked potential (MEP), n addition to the spectral power of the Delta (1-4Hz), Theta (4-8Hz), Alpha (8-12Hz), Beta (12-30Hz), Low Beta (12-20Hz), High Beta (20-30Hz) frequencies of the resting-state EEG.

Genetics analysis

 Blood was collected from patients (5 mL) in EDTA (Ethylenediaminetetraacetic acid) and DNA (deoxyribonucleic acid) was isolated by the salting-out process [16] and stored at −80◦C. The DNA samples were qualified and quantified using a NanoDrop™️ 2000 spectrophotometer (Thermo Fisher Scientific, Waltham, MA, USA). An A260/A280 ratio between 1.8 and 2.2 was used to classify the samples as high genomic DNA quality. Genotyping of OPRM1 (A118G/rs1799971 and C17T/ rs1799972) and BDNF (G196A/rs6265) polymorphisms was done by TaqMan®️ SNP Genotyping Assays (Applied Biosystems, Foster City, CA, USA). The primers and probes were predesigned assays by Applied Biosystems, and genotyping was performed on the StepOnePlus™️ instrumentation platform (Applied Biosystems, Foster City, CA, USA). Positive and negative controls were used in each genotyping assay plate, and the results of the 10% of the samples randomly selected (including positive controls) were confirmed by genome sequencing [17] in ABI 3130 Genetic Analyzer Applied Biosystems®️. Carrier status was defined as individuals carrying one or two copies of the variant allele; non-carriers as individuals homozygous for the variant allele.

Statistical analysis

Statistical analyses were performed using Stata software (StataCorp. 2021. Stata Statistical Software: Release 17. College Station, TX: StataCorp LLC). For these cross-sectional analyses, as aforementioned in the description of the instruments section, the SF-36 data were analyzed considering their division into the eight dimensions constituting this instrument (physical functioning, physical role limitations, emotional role limitations, vitality, mental health, social functioning, bodily pain, and general health perceptions), with the data from each considered as the dependent variable and those from the other instruments in the data bank as the independent variable. Notably, all variables included in the univariate analysis were selected based on a criterion and biological relevance or plausibility. Thus, only the variables that were relevant for evaluation were analyzed.

First, to ensure the normal distribution of the data, normality tests were conducted using histograms. Next, we initially ran a bivariate analysis to reveal which independent variables had a significant relationship with the SF-36 dependent variables and to determine the values ​​of the unadjusted β coefficients or odds ratios (OR) and 95% confidence intervals (CI). Next, we built the models for the multivariate analyses, based on theoretical relevance and on variables that had a p-value <0.2, thus avoiding the inclusion of potential confounding factors that did not reach the significance level of 0.05 in the univariate analysis. Variables that were not statistically significant were excluded from one of the models (backward stepwise regression), a method that is typically adopted based on the procedure’s validity in its ability to avoid suppressor effects. To guarantee the statistical quality of the models presented, the four assumptions defended by Osborne & Waters (2002)[18] were adopted: linearity, homoscedasticity, independence, and normality.

Ethical aspects

This study was approved by the Ethics Committee of the Clinics Hospital of the University of São Paulo Medical School (CAAE: 86832518.7.0000.0068). All participants signed the ICF before starting the assessments according to Brazilian research regulations (Resolution no.466) and the Declaration of Helsinki (1964) [19].

## Results

Participants

The study included 107 patients with KOA after excluding those with missing data. Among them, 83.19% were female with a mean age of 68.6 years, and an average number of days of pain was 96 days; around 31.86% were using more than five medications per day even though regarding pain measure (WOMAC and VAS), the sample showed moderate pain (WOMAC pain 10.77, VAS 5.53) with around 96 days of pain, as shown in Table 1. Regarding the ESS, the average was 10.2 (SD 5.57), which is within the normal range of sleepiness in adults. In mental health measures, the Hamilton scale showed a mean score of 9.36, suggesting mild depression. The HAD scale for depression showed 4.23 and anxiety 5.9 and none of the subscales showed a significant level of depression and anxiety. For the 10 meters walk test, the average was 11.8, and TUG mean was 15.7. Finally, relative to the assessment of OA severity, Table 2 describes the results from Kellgren-Lawrence (KL), which separately investigated the severity for each knee.

**Table 1:** Demographic aspects.

Variables		
Population (n)	113	
Gender N (%)	Male 19	16.81%
	Female 94	83.19%
Age (mean, SD)	68.6	9,4
Ethnicity N (%)	White 72	63.72%
	Brown 22	19.47%
	Black 13	11.50%
	Yellow 6	5.31%
Education N (%)	Illiterate 2	1.77%
	Fundamental 48	42.48%
	Medium 34	30.09%
	Higher 29	25.66%
Body mass index (BMI) mean (SD)	31,98	5.30
Weight mean (SD)	79.95	15.55
Height mean (SD)	1.57	. 93
Pain in days mean (SD)	96	98.7 (SD)
Conditioned pain modulation (CPM	0.87	1.21 (SD)
Pain Catastrophizing Scale mean (SD)	14.26	11.03
McGill – current intensity of pain mean (SD)	2.56	1.13
Berg balance scale mean (SD)	47.37	10.43
Medications N (%)	77	68.14
< 5 medications	36	31.86
WOMAC mean (SD)	Pain 10.77	4.18
	Stiffness 4.55	2.07
	Difficulty 35.55	14.57
	Total 48.76	21.58
Visual Analog Scale (VAS) mean (SD)	Right 5.68	2.83
	Left. 5.38	2.79
	Mediam 5.53	2.06
Epworth sleepiness scale mean (SD)	10.2	5.95
Hamilton Scale mean (SD)	9.36	5.57
Hospital Anxiety and Depression Scale (HADS) mean (SD)	Depression 4.23	3.55
	Anxiety 5.91	4.26
10 meters walk test mean (SD; min-max)	11.888	6.90079 (SD) 5.56 - 48.03 (min-max)
Timed Up and Go Test (TUG) mean (SD)	15.79	7.80
Montreal Cognitive Assessment (MoCA) mean (SD)	21.00	5.04
Transcranial Magnetic Stimulation mean (SD)	Motor-evoked potential (MEP) 1.81	1.40
	cortical silent period (CSP) 86.32	31.46
	Short-latency intracortical inhibition (SICI) .47	.27
	intracortical facilitation (ICF) 1.64	.57
	Resting Motor threshold (rMT) 51.36	11.45

**Table 2:** Kellgren-Lawrence assessment.

Kellgren-Lawrence	Classification	N	mean
Right	1	29	27.88
	2	25	24.04
	3	19	18.27
	4	31	29.81
Left	0	1	0.96
	1	33	31.73
	2	25	24.04
	3	21	20.19
	4	24	23.08

 

Considering our variable of main interest, Table 3 presents the descriptive data of the SF-36 instrument, subdivided into its eight dimensions regarding the mean values and the observed variation.

**Table 3:** Descriptive analyses of the dimensions of the SF-36.

SF-36	n	Means (±SD)	Observed variations of scale
Functional capacity	107	40.32 (22.37)	0-95
Physical aspects	107	33.87 (38.27)	0-100
Emotional	107	51.71 (45.61)	0-100
Vitality	107	53.64 (21.44)	0-95
Mental health	107	68.5 (20.73)	8-100
Social aspects	107	70.67 (28.23)	0-100
Pain	107	39.34 (23.23)	0-100
General health status	107	71.4 (16.90)	5-100

Bivariate analysis

First, bivariate analysis was performed for each domain with the following variables: WOMAC total, pain, stiffness, and difficulty, KL, BBS, TUG, six-meter walk test, 10-meter walk test, Hamilton scale, HAD scale (anxiety and depression subscale), ESS, VAS, Mcgill scale pain intensity, pain threshold, MoCA, and conditioned pain modulation (CPM). Descriptive variables were used in the bivariate analysis: gender, years of study, age, treatment time, time of pain, body mass index, and ethnicity (see supplemental material). They were tested for the bivariate model, but some were tested in the multi-model to test confounders for the analysis. Regarding neurophysiological measures in TMS, we used bivariate MEP, short-latency intracortical inhibition, and intracortical facilitation as bilateral averages. Regarding EEG measures, all analyses were performed by the frontal, central, and parietal regions to band delta, theta, alpha, beta, and gamma in resting time. It is important to highlight that for EEG and TMS variables, we did not find a correlation between any model and the SF-36. 

Multivariate analysis

As presented in the data analysis section, regarding the multivariate analysis described below, only the variables that presented the necessary criteria to be included in the models were adopted for the analyses of the respective dimensions of the SF-36. For each model, four assumptions were checked: normality, linearity, homoscedasticity, and independence.

1) Physical Functioning

Regarding functional capacity, we found that WOMAC pain (p-value 0.000), KL (p-value 0.000), TUG (p = 0.022), and anxiety (p = 0.001) were the best predictors of functional variation (approximately 51%). The Breusch-Pagan test also showed adequate homoscedasticity, and the model met the prerequisites of linearity and independence. All independent variables have a negative correlation with functional capacity.

2) Physical Role Limitations

Regarding physical aspects, WOMAC pain (p-value 0.019), ESS (p-value 0.013), and Hamilton Scale (p-value 0.003) explained approximately 24% of the variability of physical aspects in the SF-36, as in the previous domain, all correlations were negative.

3) Emotional Role Limitations

The emotional model, ESS (p = 0.016), Hamilton scale (p = 0.000), and MoCA attention score (p = 0.001) explained approximately 38% of the variability in the variable-dependent emotional domain of the SF-36. As the MoCA test could be influenced by years of study, we also used it, but it did not show any influence in this model. The final model was MoCA attention with positive relation, which means that the higher the emotional score, the better attention score.

4) Vitality

Age (p = 0.010), BBS (p = 0.005), and HAD (anxiety p = 0.000 and depression p = 0.000) better explain the vitality domain. For vitality, the HAD scale (anxiety and depression) had a negative correlation, as depression and anxiety scores were higher and lower vitality scores and physical disposition. Regarding the age and BBS scale, the relationship with vitality was positive and age seemed to act as a confounder. For this model, the independent variables explain approximately 51% of the variability of vitality. 

5) Mental Health

Anxiety (p-value 0.000), MoCA total score (p-value 0.000), and OPRM1(rs1799971)A/G polymorphism (p = 0.039)explained approximately 49% of the variability in the mental health dimension. Moreover, we ran the model with education because of its possible influence on the MoCA total score result, but it does not influence it. 

6) Social Functioning

Social aspects were related to anxiety (p = 0.000), McGill intensity of current pain (p = 0.003), and MoCA total score (p = 0.006). The final model could explain approximately 38% of the variability in social functioning, and education did not show any relevant influence. 

7) Bodily Pain 

Variability in pain dimension may also be explained by depression, measured using the Hamilton scale (p-value 0.000), ESS (p-value 0.001), VAS (p-value 0.000), and MoCA abstraction sub-scale (0.010); only this variable had a positive correlation with bodily pain. Although two of the predictors also measured some aspects of pain, in the linearity test, it was appropriate.

8) General Health Perceptions

All the correlations to general health status were negative, with higher depression and pain catastrophizing, and lower good general health perception. Regarding significance, HADS depression has a p-value of 0.000 and pain catastrophizing scale p-value of 0.001, explaining 43% of the variability in perceptions of general health. 

**Table 4:** SF-36.

Physical functioning				
Model	Coef	95% CI	p-value	Adjusted R^2^
					WOMAC pain score	-1.636.818	-2.595604 -.6780313	0.001	0.5170
KL	-6.996.634	-9.972653 -4.020616	0.000						
HAD Anxiety	-1.467.031	-2.288805 -.6452574	0.001						
TUG	-.6716169	-1.243072 -.1001618	0.022						
Physical role limitations				
WOMAC pain score	-. 4533849	-.8298109 -.076959	0. 019	0. 2477
Epworth	-1. 44236	-2.574316 -.3104034	0. 013	
HAM Depression	-1.963.565	-3.246843 -.6802883	0. 003	
Emotional role limitations				
Epworth	-1. 501819	-2.712555 -.2910824	0. 016	0. 3833
HAM Depression	-3. 846968	-5.13139 -2.562547	0. 000	
MoCa attention	7. 985343	3.34699 12.6237	0. 001	
Vitality				
Age	.4913525	.121625 .86108	0.010	0.5157
Berg balance scale	.6299413	.1986394 1.061243	0.005	
HAD Anxiety	-1.622.258	-2.516354 -.7281631	0.000	
HAD Depression	-2.352.628	-3.447365 -1.257891	0.000	
Mental health				
HAD Anxiety	-2.759.833	-3.456667 -2.062999	0.000	0. 4890
MoCa total score	1. 383863	.7835236 1.984202	0.000	
OPRM1rs1799971AG	-6.794.215	-13.22449 -.3639367	0. 039	
Social functioning				
HAD Anxiety	-2.652.627	-3.735912 -1.569343	0.000	0.3863
MoCa total score	1.299.166	.373934 2.224398	0.006	
McGill	-6.572.692	-10.8754 -2.269988	0.003	
Bodily Pain				
HAM Depression	-1. 631052	-2.209321 -1.052783	0.000	0. 5111
Epworth	-. 9177568	-1.464969 -.3705451	0.001	
MoCa abstraction	4.889.793	1.179221 8.600366	0. 010	
VAS	-3. 795258	-5.405613 -2.184904	0. 000	
General health perceptions				
HAD Depression	-2. 561834	-3.290018 -1.833649	0. 000	0. 4367
Pain catastrophizing	-3.991.101	-.6331043 -.1651159	0.001	

Inhibitory markers from TMS and EEG

All the variables indexing inhibitory activity, such as SICI and CSP, and also some EEG oscillatory frequencies such as Theta, Delta and Beta were not significantly associated with QOL either in the univariate and also multivariate analysis.

Mediation and moderation analysis

**Figure 1:**
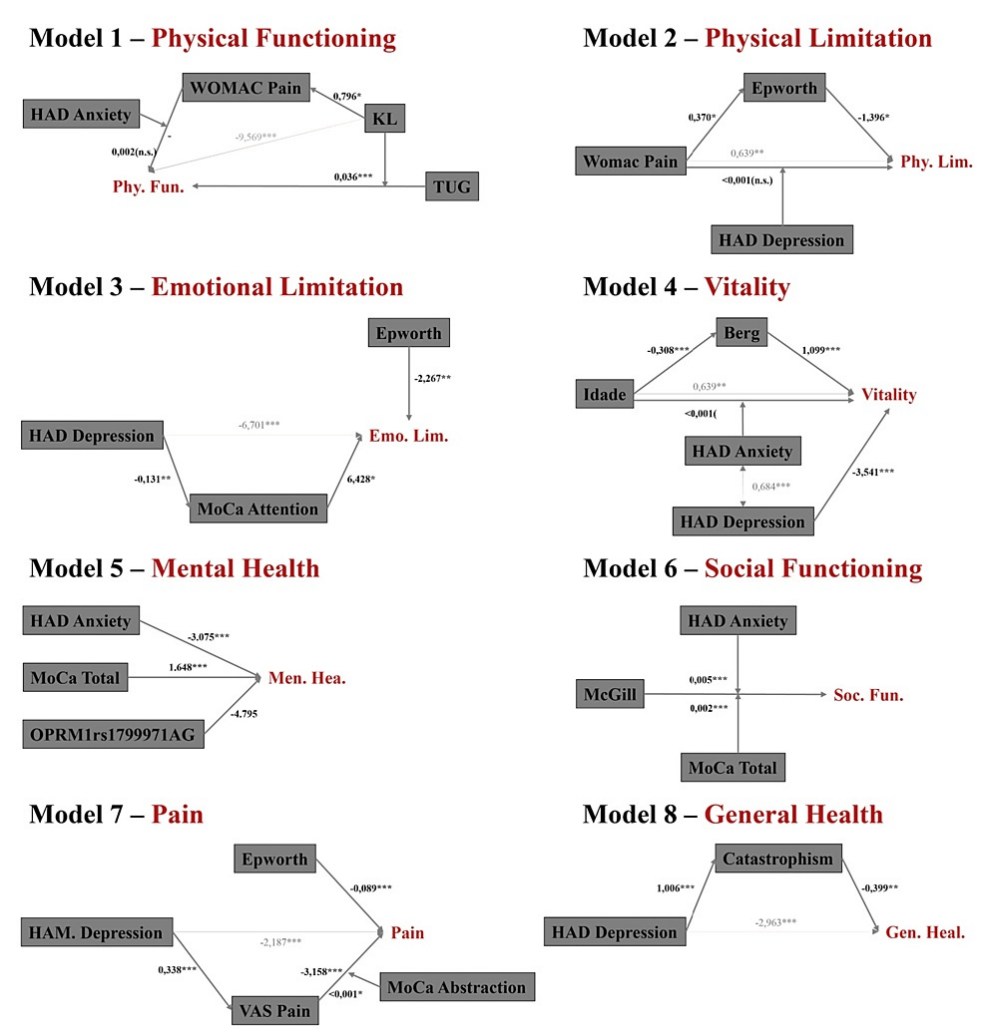
Mediation and moderation diagrams describe all eight predictive models.

From the predictive models, we created eight diagrams, one for each, in which we presented our hypotheses about how each independent variable would behave within the model. The diagrams were built from theoretical assumptions and from the results of bivariate regression analyses between all the independent variables of each model, thus allowing us to understand how the interactions took place. Figure 1 presents all the diagrams initially constructed, with significant statistical values found with the mediation and moderation analyses.

As shown in Table 5, the mediation analysis conducted showed that all the hypothesized mediations were confirmed, with the exception of the mediation WOMAC Pain → ESS → Physical Limitation (β = -0.517; SE = 0,337; LLCI = -1,337; ULCI = -0,030).

**Table 5:** Bootstrap Mediated effect for all models Percentile 95% CIs for bootstrap distributions are defined using the values that mark the upper and lower 2.5% of each distribution SE = standard error **p <0.01; ***p<0.001

									BOOSTRAP 5000 TIMES 95% CI				
	Total effect				Direct effect				Bias corrected				
	Coefficient	S.E.	t-value	p-value	Coefficient	S.E.	t-value	p-value	Effect	S.E.	Lower	Upper	Impact
Model 1 - Physical Functioning													
KL - WOMAC Pain - Physical Functioning	-9.569	1.702	-5.624	<0.001***	-7.384	1.485	-4.974	<0.001***	-2.185	1.046	-4.448	-0.385	22.80%
Model 2 - Physical Limitation													
WOMAC Pain - Epworth - Physical Limitation	-4.177	0.879	-4.754	<0.001***	-3.66	0.887	-4.126	<0.001***	-0.517	0.337	-1.337	-0.03	n.s.
Model 3 - Emotional Limitation													
HAD Depression - MoCa Attention - Emotional Limitation	-6.701	1.071	-6.255	<0.001***	-5.862	1.097	-5.342	<0.001***	-0.839	0.453	-1.858	-0.11	12.50%
Model 4 - Vitality													
Idade - Berg - Vitality	0.639	0.227	2.817	0.0058**	0.978	0.228	4.292	<0.001***	-0.339	0.113	-0.591	-0.146	-53.10%
Model 7 - Pain													
HAM Depression - VAS Pain - Pain	-2.187	0.343	-6.363	<0.001***	-1.117	0,325	-3,427	<0,001***	-1,07	0,273	-1,671	-0,587	48,90%
Model 8- General Health													
HAD Depression - Catastrophism - General Health	-2.963	0.364	-8.134	<0.001***	-2.562	0.367	-6.977	<0.001***	-0.402	0.203	-0.903	-0.11	13.60%

Finally, regarding the moderation analyses, significant moderation in the models could be observed: i) TUG moderating KL → Physical Functioning (R2-change = 0.036; F = 14.376; p = <0.001); ii) HAD Anxiety moderating McGill → Social Functioning (R2-change = 0.005; F = 37.968; p = <0.001); iii) MoCA total moderating McGill → Social Functioning (R2-change = 0.002; F = 14.130; p = <0.001); and iv) MoCA abstraction moderating VAS Pain → Pain (R2-change = <0.001; F = 4.173; p = 0.043). The other models did not show any significant interactions (Table 6).

**Table 6:** Bootstrap Moderated effect for all models **p <0.01; ***p<0.001

	Unconditional interactions				
	R2-change	F	df1	df2	p
Model 1 - Physical Functioning					
TUG - KL - Physical Functioning	0,036	14,376	1,000	109,000	<0,001***
WOMAC Pain - HAD Anxiety - Physical Functioning	0,002	0,100	1,000	109,000	0,753
Model 2 - Physical Limitation					
WOMAC Pain - HAD Depression - Physical Limitation	<0,001	0,031	1,000	109,000	0,861
Model 4 - Vitality					
Idade - HAD Anxiety - Vitality	<0,001	0,125	1,000	109,000	0,724
Model 6 - Social Functioning					
McGill - HAD Anxiety - Social Functioning	0,005	37,968	1,000	109,000	<0,001***
McGill - MoCa Total - Social Functioning	0,002	14,130	1,000	109,000	<0,001***
Model 7 - Pain					
VAS Pain - MoCa Abstraction - Pain	<0,001	4,173	1,000	109,000	0,043*

## Discussion

This study sought to explore the different predictors of HRQOL, measured using the SF-36 and subdivided into 8 main dimensions, in KOA patients from a physical rehabilitation hospital. Based on the literature, both HRQOL in patients undergoing rehabilitation and specifically in OA patients, it could be observed that several measures individually and significantly predict the dimensions of HRQOL, as well as integrated into groups, more effectively predicting such models. To discuss the vast number of results found and considering that some variables predict more than one model, we present a discussion of the results below, considering four main classes of variables: emotional, cognitive, pain-related, and functionality/vitality.

Emotional variables

Among all the independent variables analyzed, the anxiety measure was the most prevalent among the statistical models to predict the dimensions of HRQOL present in four of the eight models (physical functioning, mental health, social functioning, and vitality). Generally, higher levels of anxiety measured by the HAD instrument are related and predict lower scores of functionalities, mental health, social functioning, and vitality.

Specifically, regarding the patient's functionality, some studies show that although anxiety symptoms are not associated with physical performance, they are associated with declines in self-reported functioning, which for patients in rehabilitation is the most harmful [20]. Notably, in a recent study by our group with spinal cord injury patients (under review), it was shown that the main predictors of anxiety levels on the HAD scale were functionality characteristics such as bowel control, locomotion, social interaction, and personal hygiene. Thus, despite this study being characterized by another clinical group, it is possible to understand that, cyclically, aspects of physical functionality predict levels of anxiety and vice versa.

Regarding mental health, anxiety levels were expected to significantly predict the mental health dimension and depression levels measured by the same scale (HAD). This relationship was expected in the literature relating to these measures [21], which would make the construction of the mental health model unfeasible since the anxiety and depression variables could strictly measure the same characteristics as the mental health dimension of the SF- 36. However, we only included the anxiety measure after reviewing that both measures did not strictly assess the same aspects, thus justifying its inclusion.

Our results are consistent with the literature, which showed that anxiety is negatively related to social interaction and vitality. Despite this compatibility, the results are innovative, as they exclusively concern KOA patients. Moreover, the strong emotional aspect influences the HRQOL in this group, perhaps reinforcing the need for interventions focused on this perspective, such as those conducted [22]. Furthermore, the result of the analysis with HAD Anxiety moderating McGill → Social Functioning, demonstrates that anxiety levels can be considered as a risk factor for the impact of pain in KOA patients on social functioning.

Finally, it is important to mention that the anxiety variable was not excluded as a predictor of mental health. The main explanation is the fact that in the emotional aspects model, the anxiety of the HAD scale does not show multicollinearity and it justifies keeping with depression in the same model (vitality and general health perceptions model) because even though there is an overlap of clinical symptoms and pathophysiological processes, both are separate syndromes [23], seeing different aspects of humor symptoms. 

Regarding the variables measuring depression levels, we used two relevant measures, the HAD and Hamilton scales, to measure differences between the instruments. Moreover, we found that the HAD scale of depression is significant in predicting the dimensions of general health and vitality, and the Hamilton scale is a predictor of the dimensions of emotional aspects, physical aspects, and pain. In addition, as the mental health domain in the SF-36 approaches the impact of symptoms of depression in daily life, we expected that the multicollinearity tests for this domain would point to the model's lack of redundancy in these aspects. 

Overall, both scales predict five of the eight dimensions of HRQOL. Among the main differences between both, it can be highlighted that HAD scale can be used to measure anxiety and depression and is a valid and reliable measure of overall emotional distress, as indicated together with the SF-36 to assess patients with chronic pain [24], while the Hamilton scale is used to measure the severity of depression [25] and does not measure the extent of its effect on daily life. Generally, the correlation with the HAD and Hamilton scales could be due to the possibility of SF-36 for screening for mental disorders to identify the presence of either depression or anxiety [21].

Notably, depression levels, whether measured on HAD or Hamilton scale, predict different dimensions of HRQOL, corroborating the literature but specifically regarding KOA patients. That is, we observed in the results that the dimensions of general health perceptions, vitality, physical functioning, and emotional role limitations and that of pain in KOA patients, can be predicted by the depression levels, as observed in the healthy population. Moreover, the SF-36 could detect major depression and demonstrate a dose-effect relationship between depression type (severity) and HRQOL in chronic pain patients [26]. Nonetheless, despite the predictive character being the same in the group of KOA patients and healthy individuals, future studies can compare the differences in the depression levels between these groups and the HRQOL measured in these dimensions, possibly revealing that depression levels and different measures of HRQOL are compromised in this clinical group of KOA.

As observed for the anxiety variable HAD in the predictive model of emotional aspects and of mental health, we excluded the depression variable HAD for presenting high collinearity with anxiety HAD, but less significant for the statistical model. Even so, this variable has a high predictive value for SF-36 mental health levels, as observed in the bivariate analysis.

Both depression variables were present in the mediation model. It was observed that the predictive character of the levels of depressive symptoms in emotional role limitations, pain, and general health perceptions, were significantly mediated by the attention levels, VAS pain, and catastrophic thoughts, respectively. That is, the depression levels impact these dimensions of HRQOL through these mediators.

Finally, the last emotional variable worth highlighting its role in HRQOL models is catastrophic thinking, which significantly predicts the general health model. This finding can be explained by the fact that typically catastrophic thinking is positively related to increased intensity and exaggerated pain behavior, decreasing physical function, and prolonged disability [27]. Furthermore, catastrophic thoughts can impact behavior, both emotional and physical, leading to an impact on general health [28]. 

Cognitive variables

Our analysis revealed that the main cognitive variables, which have a predictive character, were the total levels of the MoCA instrument, predicting the dimensions of mental health and social support for HRQOL in SF-36 and the sub-aspect of attention of the MoCA instrument related to the dimension of emotional aspects and MoCa abstraction related to bodily pain model and MoCa total score for mental health and social support functioning.

Initially, regarding the total levels of the MoCA instrument, higher scores on this measure were related to greater mental health and greater social support. This result is consistent with the previous literature, as it is typically observed that, regardless of the sub-aspect, cognition levels are related to greater social adaptation and mental health [29]. The result demonstrated that total MoCA significantly mediated the predictive degree of pain, measured by the McGill instrument, in social adaptation. Second, the attention sub-aspect of the MoCA instrument positively predicts HRQOL related to emotional aspects. Generally, it is observed that lower levels of attention are related to more negative emotional processing [30], which justifies the lower levels of HRQOLto emotional aspects. We observed this impact in the mediation HAD Depression → MoCA Attention → Physical Limitation, in which attentional levels significantly mediated the predictive degree of depression levels in emotional limitation.

In all four statistical models, which included the MoCA instrument variables, we also included the education variable to reveal or control the possible confounding impact of education on the predictive value of cognition under the HRQOL. Thus, education was included in the initial mental health, social functioning, emotional role limitations, and pain models. Education does not appear as a confounder but contaminated the model, not contributing to the predictive value as a whole and decreasing the weight of the contribution of variables such as total MoCA levels and anxiety levels; hence, it was excluded from the final model. 

We also found a significant impact of MoCA abstraction in the bodily pain model, explaining the cognitive face of pain that is widely known, in which pain tends to guide poorer cognitive functioning, even though it was found in domains of attention and executive function of MoCA, not in abstraction [31]. Our moderation analysis with levels of abstraction impacting the predictive degree of VAS pain in the pain dimension of the SF-36 indicates that the cognitive ability to abstract can be considered a protective factor for KOA patients. It separates from the sensation of pain and reduces the impact on their HRQOL.

Pain-related variables

Regarding the related pain measures, pain catastrophizing is widely known in the health behaviors impacting physical activities, and the fear-avoidance caused by them might result in slowing the rehabilitation process and becoming a cycle of pain-related fear, impairment, and disability. Nonetheless, pain is a floating measure and context-specific, which makes it more important for factors that could contribute to daily fluctuations in joint pain among patients with KOA [32]. Regarding the intensity of pain measured using VAS, the correlation with bodily pain was negative, which is understandable since pain intensity negatively impacts HRQOL, similar to the findings by Bostrom et al. [33], who observed different levels of pain in cancer patients.

Regarding CPM, a significant measure related to endogenous pain-inhibitory capacity, it does not have a predictive impact in any model, even in bivariate analysis, which may be because the biggest part of our sample efficiency responds to it (pain threshold variation greater than 10%). In KOA, these variables will be more important when we perform longitudinal analysis where we expect that patients with higher CPM at baseline may show a higher reduction in pain after treatment or rehabilitation, as seen in another study [34].

Functional/vitality variables

Regarding the functional and vitality domains, the independent variables understood that ESS, age, BBS, and KL, assess aspects that could affect execution. Previous studies [35] have shown a correlation between vitality and functional independence in KOA patients. Regarding correlation with ESS, it is recognized that sleep disturbance is common among patients with osteoarthritis [36]. Studies were conducted to treat insomnia for pain improvement [37], and we should be cautious when treating patients with problems in central pain modulation. In older adults, it may be worse because the association with poor sleep is high [38] and which could explain ESS as a predictor of physical and bodily pain in our study. 

Considering the BBS, our predictive results may be consistent with the literature, since it contributes to mobility limitations and difficulties in daily life [39]. The same could be said for KL, with a negative correlation in physical functioning, which is consistent with the literature [40]. Furthermore, our moderation analysis demonstrated that the degree of injury (KL) significantly affected how TUG performance predicted physical functionality. Another interesting result of these moderation analyses was that balance levels (BBS) significantly moderate the predictive degree of age on vitality in KOA patients.

Genetic polymorphism

The last topic relevant to this discussion concerns the genetic polymorphism of OPRM1/rs1799971. As aforementioned, the data presented in this paper are part of a large, ongoing study, described in detail by Simis et al. [15], which uses behavioral and biomarker measures and genetic polymorphism to explore predictors of potential inhibitory deficits in patients undergoing rehabilitation. Genetic polymorphism reveals the significant relationship of only the genetic polymorphism OPRM1/rs1799971, but not the other polymorphisms (OPRM1/rs1799972 and BDNF/rs6265). Specifically, the genetic polymorphism measure of OPRM1/rs1799971 in the individual regression analyses presented a predictive character for the mental health dimension, remaining significant even within the model created with the anxiety and MoCA total variables (together explaining approximately 49% of the variation in the SF-36 Mental Health). It has been observed that OPRM1/rs1799971 is involved in reward and analgesic pathways [41] and plays a role in cognitive dysfunction [42].

Neurophysiological biomarkers

The neurophysiological measures (transcranial magnetic stimulation, TMS; electroencephalography, EEG spectral analysis) in this dataset were collected to be tested as predictors of other dependent variables, such as pain and motor (as described in Simis et al. [15]). Our initial hypothesis did not include the SF-36 because of the lack of clear functional anatomy correlations between HRQOL and specific cortical activities. However, due to evidence that EEG measures were correlated with social and mental aspects [43], neurophysiological measures were tested in this study. The results of these analyses were not significant, as our initial hypothesis, and it might be because of the absence of pathological correlation between biomarker measures and the HRQOL constructs verified in the SF-36.

Strengthens and Limitations

The strengths of this study are its large number of patients compared with other similar studies and the use of different instruments to measure the same aspects, such as pain and depression, which makes the analysis more complex and appropriate for modeling a construct multicausal as HRQOL. A limitation is that it did not include patients’ information before rehabilitation, such as data on anxiety and depression.

## Conclusions

This work revealed the main predictors of eight aspects of HRQOL in KOA patients in a physical rehabilitation hospital. Among the main relevant variables, emotional aspects such as anxiety and depression were present in all evaluated dimensions. Cognitive variables were significant, specifically regarding mental health, emotional aspects, and social support dimensions. As expected, pain-related variables predominantly predict the HRQOL regarding functionality and patient pain. Finally, variables related to the functionality and vitality of patients significantly predicted all dimensions studied, except for mental and general health. To the best of our knowledge, this is the first study to describe physical, cognitive, pain-related, and functional predictors of HRQOL in patients with KOA. The results contribute to the general literature on physical rehabilitation and are specific to this clinical group. Moreover, our study added findings with polymorphism regarding the mental aspect, predicting the dimension of the SF-36, which strengthens the study by including an objective measure among the many subjective ones.
